# Distribution, expression of hexaploid wheat *Fes1s* and functional characterization of two *TaFes1As* in Arabidopsis

**DOI:** 10.3389/fpls.2022.1037989

**Published:** 2022-10-17

**Authors:** Yunze Lu, Mingran Ha, Xinming Li, Junzhe Wang, Ruirui Mo, Aihua Zhang

**Affiliations:** ^1^ School of Landscape and Ecological Engineering, Hebei University of Engineering, Handan, China; ^2^ State Key Laboratory of Crop Stress Biology for Arid Areas, College of Agronomy, Northwest A&F University, Yangling, China

**Keywords:** hexaploid wheat, Fes1, thermotolerance, evolution, expression

## Abstract

Hexaploid wheat is a major food crop and is sensitive to heat stress. It is necessary to discover genes related to thermotolerance in wheat. Fes1s is a class of nucleotide exchange factor of heat shock protein 70s, proven to be participated in heat response in human, yeast, and Arabidopsis. However, little is known about Fes1s in hexaploid wheat. In this study, we identified nine *Fes1s* in hexaploid wheat (*TaFes1s*) and found that they present as three triads. A phylogenetic relationship analysis revealed that these *Fes1s* grouped into *Fes1A*, *Fes1B* and *Fes1C* subclades, and *Fes1As* and *Fes1Bs* were divergent in monocots, but possibly not in dicots. The sequences, gene structures and protein motifs of *TaFes1s* homoeologues within a triad were highly conserved. Through cis-elements analysis including heat shock elements, and miRNA targets prediction, we found that regulation of three *TaFes1s* homoeologues may be different, while the expression patterns of three homoeologues were similar. The expression levels of *TaFes1As* were higher than those of *TaFes1Bs* and *TaFes1Cs*, and based on these expressions, *TaFes1As* were chosen for functional characterization. Intriguingly, neither *TaFes1A-5A* nor *TaFes1A-5D* could not rescue the thermotolerance defect of Arabidopsis *fes1a* mutants at seedling stage, but in the transgenic plants seed germination was accelerated under normal and heat stress condition. The functional characterization indicated that roles of *Fes1As* would be different in Arabidopsis and hexaploid wheat, and function retention of *TaFes1As* may occur during wheat evolution. In conclusion, our study comprehensively characterized the distribution and expression of *Fes1s* in hexaploid wheat and found that two *TaFes1As* could accelerate seed germination under normal and heat stress condition.

## Introduction

Hexaploid wheat (*Triticum aestivum* L.) is a staple crop that accounts for about 28% of global cereal production, and it is vulnerable to heat stress (Tack et al., 2015; Yadav et al., 2022). Heat stress significantly impedes the production and quality of wheat, and when above the optimum temperature, each increase of a single degree could reduce wheat yield by 5-10% (Liu et al., 2014; Lesk et al., 2016; Zhao et al., 2017). With global warming, the global mean temperature is predicted to increase 1.5°C in the next two decades, and together with more frequent extreme and short-term high temperature, heat stress will become a major abiotic factor that threatens wheat production (Liu et al., 2014; Tack et al., 2015; Zhao et al., 2017; Yadav et al., 2022).

As a sessile organism, wheat is vulnerable to numerous unfavorable environmental conditions, such as heat stress, thus, wheat has evolved complicated adaptive mechanisms at molecular level to cope with heat stress. Under heat stress, many proteins become misfolded and aggregate in cells disturbing plant growth and development, hence, the chaperone system involved in protein folding and quality control of unfolded proteins is activated (Wang et al., 2004; Buchberger et al., 2010). In this chaperone system, the heat shock protein 70 (HSP70) system plays a pivotal role, participating in protein synthesis, folding, transportation, translocation, activity regulation, and the prevention of aggregation (Wang et al., 2004; Buchberger et al., 2010; Rosenzweig et al., 2019). Characterization and expression of *HSP70s* in hexaploid wheat have been reported (Kumar et al., 2020; Lu et al., 2022).

The HSP70 chaperone system includes HSP70, co-chaperone HSP40 (DnaJ), and a nucleotide exchange factor (NEF). Each component has its own role: HSP70 acts as the chaperone interacting with its substrates; HSP40 directly interacts with HSP70 and mediates the substrates binding to HSP70 in synergism with ATP hydrolysis on HSP70, leading to the trapping of the substrate. NEF promotes the dissociation of ADP and the rebinding of ATP on HSP70, consequently resulting in substrate release from HSP70. Thus, binding and releasing of substrates in the HSP70 system are tightly related to the ADP/ATP exchange cycle, in which the dissociation of ADP is a rate-limiting step (Bukau and Horwich, 1998; Mayer and Bukau, 2005; Kampinga and Craig, 2010; Rosenzweig et al., 2019).

In eukaryotic cytosol, three classes of HSP70 NEFs are found, including Armadillo families (HSPBP1 in human, Fes1p in yeast), Bcl2-associated athanogene domain families, and HSP110 (Bracher and Verghese, 2015; Rosenzweig et al., 2019). HSPBP1 is thought to determinate the fate of substrate to degradation or not (Alberti et al., 2004). Fes1p is essential to the degradation of misfolded proteins in yeast (Gowda Naveen Kumar et al., 2013; Gowda et al., 2016). In Arabidopsis, three Fes1p homologues are characterized (AtFes1A, AtFes1B, AtFes1C); AtFes1A localizes in cytosol and interacts with HSP70, and the loss of *AtFes1A* leads to thermosensitivity of Arabidopsis plants (Zhang et al., 2010). AtFes1A is also required for the function of the Arabidopsis molecular chaperone system (Fu et al., 2015). In rice, three Fes1p homologues are identified (OsFes1A, OsFes1B, OsFes1C); in contrast to OsFes1A and OsFes1B which are located in cytosol and nucleus, OsFes1C is an ER-localized protein, and OsFes1C is involved in ER and salt stress (Qian et al., 2021). Overexpression of Seagrass *Fes1* could improve the thermotolerance of transgenic Arabidopsis plants (Chen and Qiu, 2020). However, the distribution and roles of *Fes1s* in hexaploid wheat are still unknown.

Hexaploid wheat is derived from two major inter-specific hybridizations. The first hybridization occurs between two diploid species, *T. urartu*, and a possibly extinct *Aegilops* species closely related to *Ae. speltoides*. This hybridization forms a tetraploid wheat, wild emmer wheat (*T. turgidum* ssp. *Dicoccoides*). A second hybridization between wild emmer wheat and *Ae. tauschii* finally generates hexaploid wheat. Thus, the hexaploid wheat comprises three subgenomes (AA, BB, and DD), and many genes have copies/pairs distributed in these subgenomes, termed homoeologues (Dubcovsky and Dvorak, 2007; Marcussen et al., 2014; Levy and Feldman, 2022). The homoeologous genes has a single copy on each subgenome are regarded as a triad (Appels et al., 2018).

In this study, we first analyzed the distribution of *Fes1s* in hexaploid wheat (*TaFes1s*) and its progenitors. Then, the sequence characteristics and cis-elements of *TaFes1s* were identified, as well as their expression profiles in hexaploid wheat during growth and under stress condition. Finally, *TaFes1A-5A* and *TaFes1A-5D* were transformed into the Arabidopsis thermosensitive *fes1a* mutant, and we found that overexpression of both genes accelerated the germination of transgenic seeds under normal and heat conditions, rather than inhibiting the thermosensitivity of *fes1a* mutants. Our results indicate that the roles of *Fes1As* may be different in hexaploid wheat and Arabidopsis.

## Materials and methods

### Characterization of *Fes1* genes in hexaploid wheat and its progenitors

Genome and protein sequences of hexaploid wheat (*T. aestivum*), *T. urartu*, *Ae. tauschii*, *T. turgidum* ssp. *Dicoccoides* were downloaded from Ensemblplants database, sequences of *Ae. speltoides* were downloaded from the e!DAL databse (Avni et al., 2022). Those sequences comprised the database in blastp program, and Fes1 protein sequences from Arabidopsis and rice were used as a query to search against the database above, with the following criteria: identity >50% and e value <1e-5. Besides, the Hmmsearch engine in the HMMER3.0 program was used to search the same database using the HMM profile of Fes1 domain (PF08609 in the Pfam database) as a query, with the threshold of 1e-5. The results of blastp and Hmmsearch were merged and manually corrected, and then subjected to NCBI CDD database to confirm the presence of the Fes1 domain. Those proteins containing Fes1 domain were characterized as Fes1 proteins.

### Phylogenetic analysis of Fes1 proteins

All the Fes1 proteins were aligned in the MAFFT program with the “L-INS-i” algorithm, and then the aligned sequences were submitted to IQ-tree to construct a maximum likelihood tree with 1000 bootstrap replicates with the command “-m MFP -b 1000 -redo -nt AUTO” (Nguyen et al., 2015). A substitution model of JTT+G4 was selected based on the Bayesian information criterion in the ModelFinder (Kalyaanamoorthy et al., 2017). The tree file was visualized *via* Figtree v1.4.4.

### Gene structures, protein motifs, cis-elements, miRNA targets analysis of Fes1s in hexaploid wheat

For convenience, we renamed the *Fes1* genes in hexaploid wheat as *TaFes1X-YZ*, in which *Ta* means *T. aestivum*, *X* represents the phylogenetic clade and *YZ* refers to the chromosome localization. For example, *TaFes1A-5A* means this wheat *Fes1* gene was grouped in Fes1A clade and localized in chromosome 5A. Gene structures of *TaFes1s* were obtained from the genome annotation GFF3 files. Protein sequences of TaFes1s were submitted to the MEME to identify a maximum of 12 motifs ranging from 6 to 200 aa, other parameters in MEME were set as default. Finally, the gene structures and protein motifs were illustrated by TBtools (Chen et al., 2020).

The 2-kb genomic sequence from the transcription initiation site of each *TaFes1* was extracted and searched for cis-elements in the PlantCARE database (Lescot et al., 2002). Heat shock elements (HSEs) were identified as described (Zhao et al., 2020; Lu et al., 2022). Briefly, the pentanucleotide motifs 5’-NGAAN-3’ and 5’-NTTCN-3’ were regarded as a unit in which N represent any nucleotide, and the unit numbers must be continuous at least three, these HSEs were regarded as typical HSEs; for varied HSEs, only one mismatch was allowed but mismatch was not permitted to be occurred on the “G” of 5-NGAAN-3 (“C” for 5’-NTTCN-3’) in the first and last unit if the unit number was three. To predict the *TaFes1s* targeted by microRNAs (miRNAs), all the *TaFes1s* transcripts were subjected to psRNATarget tool (Dai et al., 2018) to searched against the published wheat miRNAs with default parameters.

### Expression analysis of *TaFes1s*

The transcription abundances of *TaFes1s* in wheat varieties “Azhurnaya” and “Chinese Spring” under normal conditions were obtained from a previous study (Ramírez-González et al., 2018), expression profiles of *TaFes1s* under heat and drought stress were obtained from other literatures (Liu et al., 2015; Wang et al., 2019; Ma et al., 2021).

Two *TaFes1s* were selected for qPCR validation (**Supplementary Table 1**). One week old seedlings of “Chinese Spring” and “TAM107” were subjected to drought stress (mimic by 20% PEG6000), heat stress (40°C), and combined heat and drought stress for 1 and 6 hours as described (Liu et al., 2015). RNA was isolated from leaves and about 1 μg total RNA was reverse-transcribed using the PrimeScript™ RT reagent Kit with gDNA Eraser (Perfect Real Time) (Takara, Dalian, China), and qPCR was performed on a Thermo Fisher Scientific QuantStudio 3 Real-Time PCR System using the TB Green^®^ Premix Ex Taq™ II (Tli RNaseH Plus) kit (Takara, Dalian, China). RNA isolation and qPCR experiments were performed by three independent biological replicates.

### Gene cloning, plasmid recombination, plant transformation

The CDSs of *TaFes1A-5A* and *TaFes1A-5D* were individually amplified (**Supplementary Table 1**) and transformed into pBIB-35S-Hygromycin-*gene*-GFP to construct a recombined vector *via* Gateway cloning technology, respectively. The recombined vector was confirmed by sequencing. Then the recombined vector was transformed into Agrobacterium GV3101. Finally, the recombined vector was transformed into Arabidopsis *fes1a* mutant by the floral dipping method.

### Plant growth and heat stress treatment

Surface sterilized seeds of three independent T_3_ homozygous transgenic lines were planted on 1/2 MS media and kept at 4°C for three days. Then the plates were transferred to growth chamber (35/19°C, 16/8h) to evaluate the thermotolerance of seeds at germination stage. The germination rate was calculated as the ratio of germinated seeds to total seeds under both normal and heat stress condition (22/19°C, 16/8h). All plates were photographed every day. All experiments were repeated at least three independent times.

## Results

### Evolution of Fes1s in several monocots and dicots species

**Figure 1:**
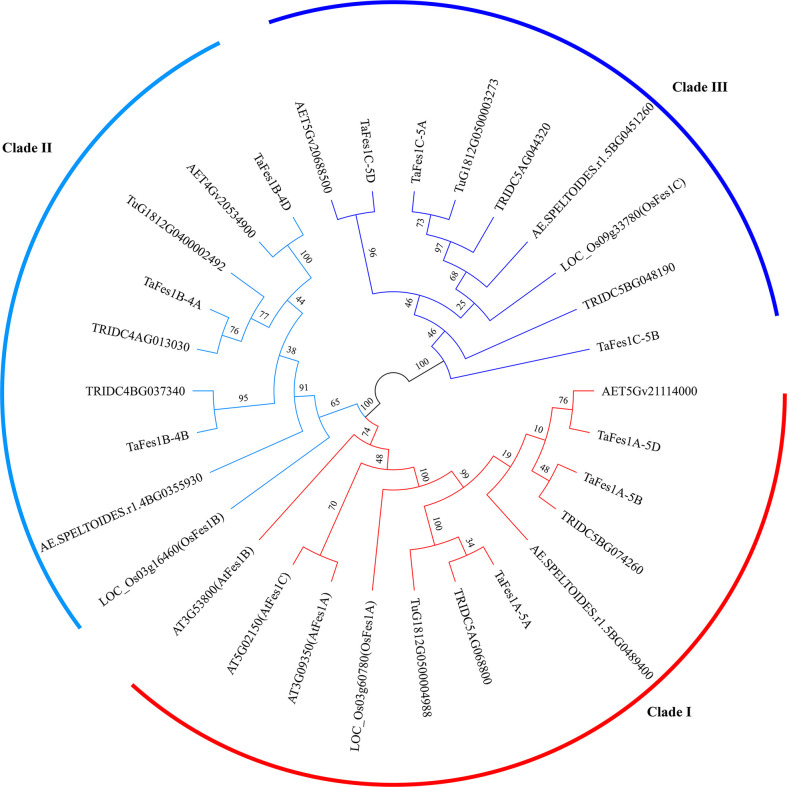
Phylogenetic relationship of *Fes1s* in hexaploid wheat and its progenitors. The maximum likelihood tree is constructed by IQ-tree software and evaluated by 1000 bootstrap replicates. Numbers near branches represent the percentage of 1000 bootstrap values. Different clades of Fes1s are represent by different colors. Species names are embedded in the *Fes1s* geneIDs: AET, *Ae. tauschii*; Tu, *T. urartu*; AE.SPELTOIDES, *Ae. speltoides*; TRIDC, *T. turgidum* ssp. *Dicoccoides*; Ta, *T. aestivum*; LOC, *Oryza sativa* ssp. *Japonica*; AT, *Arabidopsis thaliana*.

Using the blastp and Hmmsearch engine, three *Fes1s* were identified in each diploid species (*T. urartu*, *Ae. tauschii*, *Ae. speltoides*), and six and nine *Fes1s* were characterized in wild emmer wheat and hexaploid wheat, respectively; thus, the copy number of *Fes1s* in hexaploid wheat and its progenitors was consistent with the ploidy level. Using Arabidopsis and rice *Fes1s* as marker, the *Fes1s* in monocots were evenly and clearly classified into *Fes1A*, *Fes1B*, and *Fes1C* (**Figure 1**). Interestingly, the Arabidopsis *Fes1s* were all grouped in Clade I in which the *Fes1s* of monocots were present. To detect the relationship of *Fes1s*, we characterized the *Fes1As* in more monocots and dicots species. Based on the phylogenetic tree constructed by maximum likelihood (**Supplementary Figure 1**), except for the *Fes1s* in Arabidopsis, *Fes1Cs* evolved into a separate clade in both monocots and dicots, and *Fes1As* and *Fes1Bs* were also divided into different clades in monocots, while *Fes1As* and *Fes1Bs* remained in a clade and possibly were not divergent in dicots.

The *Fes1s* in hexaploid wheat present as three triads, and only occurred on chromosome group 4 and 5 (**Table 1**). All *TaFes1s* on chromosome 5 were distributed in distal telomeric regions (R3), and those on chromosome 4 were found in the interstitial regions (R2a and R2b). The protein length of TaFes1As were the shortest.

**Table 1:** Information of *TaFes1s*.

Name	GeneID	MW (kD)	Length (aa)	Gene length	Intron number	Splice variant	Chromosome segment
*TaFes1A-5A*	TraesCS5A02G482400	41.08	381	4210	5	2	R3
*TaFes1A-5B*	TraesCS5B02G495500	40.94	381	4061	5	2	R3
*TaFes1A-5D*	TraesCS5D02G496000	40.86	381	3904	5	2	R3
*TaFes1B-4A*	TraesCS4A02G095500	43.79	399	2855	4	2	R2a
*TaFes1B-4B*	TraesCS4B02G208900	45.44	410	3805	4	4	R2b
*TaFes1B-4D*	TraesCS4D02G209700	45.53	412	2751	4	2	R2b
*TaFes1C-5A*	TraesCS5A02G296200	44.38	412	3902	7	1	R3
*TaFes1C-5B*	TraesCS5B02G295400	45.03	416	3991	7	1	R3
*TaFes1C-5D*	TraesCS5D02G303400	44.58	412	4139	7	1	R3

### Gene structures, protein motifs analysis of *TaFes1s*

**Figure 2:**
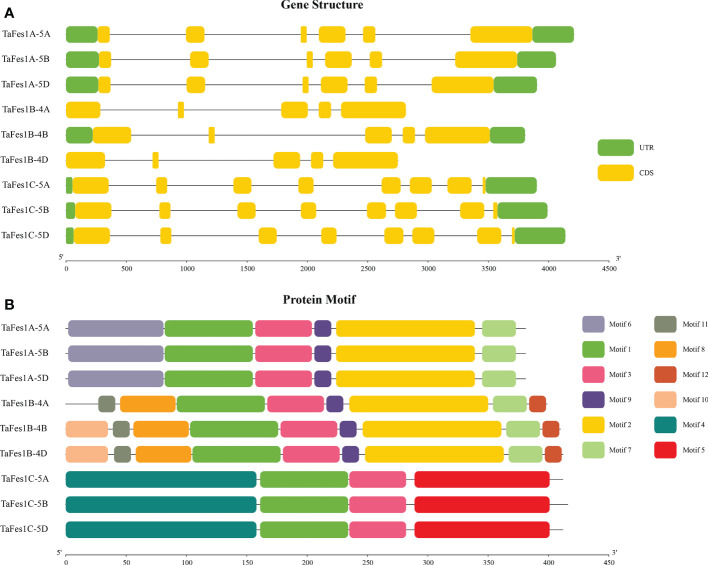
Gene structures and protein motifs of TaFes1s. **(A)** Gene structures of *TaFes1s*. Green boxes represent untranslated regions (UTR), yellow boxes refer to coding sequences (CDS), lines mean introns. The scale in bottom indicates gene length in base pairs unit. **(B)** Protein motifs of TaFes1s. Motifs are identified by MEME. Different motifs are shown in different colors, lines are protein sequences which none conserved motif is characterized. The scale in bottom indicates protein length in amino acid unit.

Gene structures and protein motifs could explain the phylogenetic relationship of a gene family to some extent (Zhu et al., 2020). Further analysis found that the gene structures of each *TaFes1* homoeologue were conserved in a triad but different among triads (**Figure 2A**). The intron numbers were five, four, and seven for homoeologues in *TaFes1As*, *TaFes1Bs*, and *TaFes1Cs*, respectively. However, the splice variants were somehow not consistent with the intron numbers. All *TaFes1As* and *TaFes1Cs* generated two and one variants. While one homoeologue of *TaFes1Bs*, *TaFes1B-4B*, had 4 variants and the other two had 2 variants.

The protein motifs of each homoeologue were also conserved in a triad but different among triads (**Figure 2B**, **Supplementary Table 2**). Six, eight to nine, and four motifs were identified in TaFes1As, TaFes1Bs, and TaFes1Cs, respectively. Among all the twelve identified motifs, only Motifs 1 and 3 were shared by all TaFes1s. Besides, Motifs 9, 2, and 7 were common amongst TaFes1As and TaFes1Bs. This was consistent with the finding in the phylogenetic tree that Fes1As and Fes1Bs were more closely related. In addition, triad-specific motifs were also characterized, and they were Motif 6 in TaFes1As, Motifs 11, 8, 12 and 10 in TaFes1Bs; and Motifs 4 and 5 in TaFes1Cs. The gene structures and protein motif analyses indicate that the function of TaFes1s may be conserved within triads but distinct among triads.

### Cis-elements and miRNA targets analysis of *TaFes1s*

**Figure 3:**
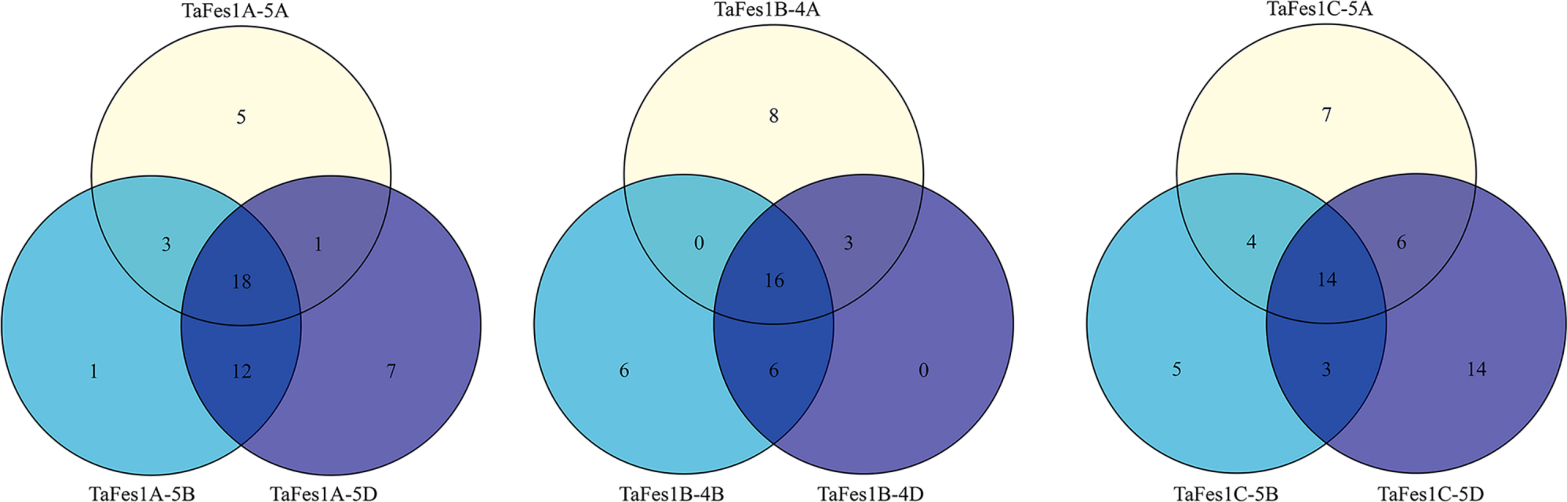
Comparison of cis-elements in *TaFes1* homoeologues.

Cis-elements are important regulatory factors participating in the transcriptional regulation of genes during plant growth, development and stress response. The upstream 2-kb sequences of the nine *TaFes1s* were extracted for cis-elements analysis. At least 57 elements were identified and assigned into three groups: Hormone signaling, Abiotic/Biotic stress, and Others (**Table 2**). The five most common (total number in all Fes1s) were: TGACG-motif (MeJA responsiveness), CGTCA-motif (MeJA responsiveness), ABRE (Abscisic acid responsiveness), TCA-element (salicylic acid responsiveness), and AuxRR-core (Auxin responsiveness) in “Hormone signaling” group; STRE, G-box, as-1, ARE, and Sp1 in “Abiotic/Biotic stress” group; CAAT-box, TATA-box, MYB, MYC, CCGTCC motif in “Other” groups. Among the 57 elements, only six (CAAT-box, MYB, STRE, as-1, CGTCA-motif, TGACG-motif) were present in all *TaFes1s*. Intriguingly, the TATA-Box was absent in *TaFes1B-4A*. In each triad, each homoeologue except *TaFes1B-4D* contained specific elements (**Figure 3**), indicating that the expression profiles of *TaFes1* homoeologues may be different.

**Table 2:** Distribution of cis-elements in promoters of *TaFes1s*.

Gene	Hormone signaling	Abiotic/Biotic stress	Others
*TaFes1A-5A*	ABRE,CGTCA-motif,TGACG-motif,TCA-element,P-box	as-1,G-box,GC-motif,Sp1,WUN-motif,ARE,ATCT-motif,box S,DRE core,GT1-motif,I-box,LTR,STRE,TCCC-motif,WRE3	CAT-box,CAAT-box,TATA-box,MYC,AAGAA-motif,MYB
*TaFes1A-5B*	ABRE,CGTCA-motif,TGACG-motif,GARE-motif,P-box,TCA-element	as-1,Sp1,ARE,G-box,Box 4,DRE core,GC-motif,STRE,WRE3,3-AF1 binding site,3-AF3 binding site,GT1-motif,LTR,WUN-motif	dOCT,CAT-box,CCGTCC motif,CAAT-box,TATA-box,MYB,A-box,AT-rich element,MYC
*TaFes1A-5D*	ABRE,CGTCA-motif,TGACG-motif,GARE-motif,O2-site,TCA-element	as-1,ARE,G-box,AAAC-motif,Box 4,GC-motif,MBS,Sp1,STRE,WRE3,3-AF1 binding site,3-AF3 binding site,box S,chs-Unit 1 m1,DRE core,GT1-motif,LTR,MRE,W box	CCGTCC motif,dOCT,F-box,CAAT-box,TATA-box,MYB,MYC,A-box
*TaFes1B-4A*	TCA-element,ABRE,CGTCA-motif,TGACG-motif	as-1,ACE,ARE,DRE core,GC-motif,Sp1,TCCC-motif,WRE3,CCAAT-box,G-box,STRE	CAT-box, CCGTCC motif,MYB,A-box,MYB recognition site,MYC,CAAT-box
*TaFes1B-4B*	TCA-element,AuxRR-core,CGTCA-motif,ERE,TGACG-motif	as-1,STRE,LTR,box S,TCCC-motif,ACE,Box 4,DRE core,GC-motif,TCT-motif,W box,WRE3,WUN-motif	CCGTCC motif,GCN4_motif,CAAT-box,TATA-box,MYB,AAGAA-motif,CTAG-motif,A-box,MYC
*TaFes1B-4D*	TCA-element,AuxRR-core,CGTCA-motif,TGACG-motif,ABRE	as-1,STRE,box S,TCCC-motif,ACE,DRE core,G-box,GC-motif,LTR,Sp1,WRE3	CCGTCC motif,CAAT-box,TATA-box,CTAG-motif,AAGAA-motif,MYB,MYC,A-box
*TaFes1C-5A*	CGTCA-motif,TGACG-motif,ABRE,O2-site,TCA-element	as-1,STRE,GC-motif,I-box,WRE3,WUN-motif,ACE,ARE,ATCT-motif,CCAAT-box,G-box,LAMP-element,MBS,TCT-motif	CCGTCC motif,CAAT-box,TATA-box,A-box,MYC,AAGAA-motif,MYB,MYB recognition site
*TaFes1C-5B*	ABRE,AuxRR-core,CGTCA-motif,P-box,TGACG-motif	as-1,STRE,G-box,AE-box,MBS,WRE3,box S,I-box,Sp1,TCCC-motif,TCT-motif,W box	CAT-box,CCGTCC motif,CAAT-box,TATA-box,MYB,MYC,AAGAA-motif,A-box
*TaFes1C-5D*	CGTCA-motif,TGACG-motif,ABRE,CARE,ERE,GARE-motif,JERE,O2-site,TCA-element,TGA-element	as-1,G-box,STRE,DRE core,GT1-motif,TCT-motif,W box,AE-box,box S,CCAAT-box,DRE1,GATA-motif,I-box,LTR,MBS	CCGTCC motif,CAAT-box,TATA-box,MYB,MYC,A-box,MYB recognition site

However, none typical HSE elements were identified in all *TaFes1s via* PlantCARE database. This was somehow confused because that Fes1s were the nucleotide exchange factors of the important heat responsive genes, *HSP70s*, *TaFes1s* were possibly heat responsive. Using our previous procedure, we first analyzed HSEs in the upstream 2 kb sequences of *TaFes1s*. Consistently, none typical HSEs were characterized in all *TaFes1s*, and four varied HSEs were identified in four *TaFes1s* (**Table 3**). Longer promoter sequences were further extracted and analyzed, and this resulted in 13 more HSEs being identified.

**Table 3:** HSEs in *TaFes1s* promoters.

No.	Gene	Position tart^1^	Position End^1^	HSE_seq^2^	Mismatch	Analyzed sequence length^3^
1	*TaFes1A-5A*	-331	-317	GGCACATTCCAGAAA	CA	2000
2	*TaFes1B-4B*	-1527	-1513	TTTCTAGCACTTTCT	CA	2000
3	*TaFes1C-5A*	-19	-5	CATCTCGAAAATTCC	AT	2000
4	*TaFes1C-5D*	-1985	-1971	GGACCATTCCGGAAC	CA	2000
5	*TaFes1A-5A*	-4070	-4056	TTACACGAACATTCT	AT	5263 (263)
6	*TaFes1A-5A*	-594	-580	GGCACATTCCAGAAA	CA	5263 (263)
7	*TaFes1A-5B*	-2415	-2401	AGAGTTTTCAGGAAG	GA	5274 (274)
8	*TaFes1A-5B*	-240	-226	CGCAGGTTCCGGAAC	CA	5274 (274)
9	*TaFes1A-5D*	-233	-219	CGCAGGTTCCGGAAC	CA	3108 (269)
10	*TaFes1B-4B*	-4738	-4724	ATTCAAGAAAAATCT	AT	5222 (222)
11	*TaFes1B-4B*	-1749	-1735	TTTCTAGCACTTTCT	CA	5222 (222)
12	*TaFes1B-4D*	-3759	-3745	TGAATGATCAAGAAC	AT	4741 (0)
13	*TaFes1C-5A*	-73	-59	CATCTCGAAAATTCC	AT	5054 (54)
14	*TaFes1C-5B*	-71	-57	CATCTCGAAAATTCC	AT	5076 (76)
15	*TaFes1C-5D*	-4853	-4839	TTTCATGAAGTATCC	AT	5064 (64)
16	*TaFes1C-5D*	-2049	-2035	GGACCATTCCGGAAC	CA	5064 (64)
17	*TaFes1C-5D*	-70	-56	CATCTCGAAAATTCC	AT	5064 (64)

^1^The last nucleotide in the 3’ end was regarded as “-1”, thus the position was showed as the distance from the 3’ end.

^2^The mismatch nucleotide in the HSE element was underlined.

^3^In this column, sequences in 2000 mean the upstream 2-kb sequences from the transcription initiation site were obtained for HSE analysis; Sequences in other length mean the upstream 5-kb sequences from the transcription initiation site sequences, together with sequences from the transcription initiation site to the start codon (length marked in bracket), were extracted for HSE analysis. For TaFes1A-5D and TaFes1B-4D, sequences were shorter because of sequencing gaps in the genome sequences (tandem N).

**Figure 4:**
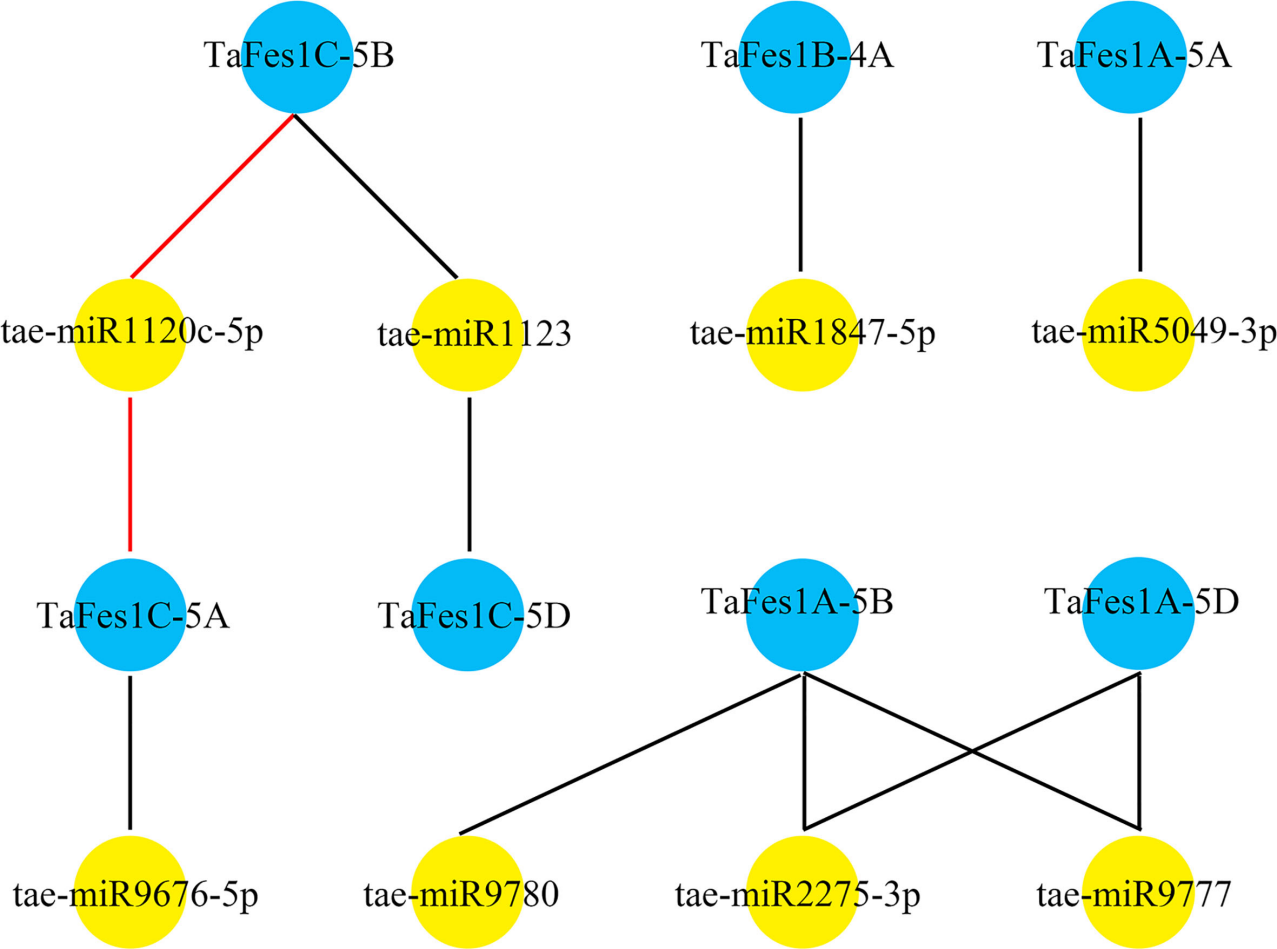
Potential regulation association between miRNAs and *TaFes1s*. MiRNAs are represented by yellow circles and *TaFes1s* are represent by blue circles. Dark lines link miRNAs and *TaFes1s* indicate the regulation relationship are transcription cleavage, and red lines are translation inhibition.

MiRNAs are a widespread class of non-coding regulatory endogenous RNA with 20-22 nucleotides in length, and they act as critical posttranscriptional regulators in gene expression, being involved in plant growth and stress response (Khraiwesh et al., 2012; Pagano et al., 2021). The putative regulatory association between *TaFes1s* and miRNAs was predicted by psRNAtarget tool, and seven *TaFes1s* were predicted to regulated by eight miRNAs (**Figure 4**). Only one miRNA silenced *TaFes1s via* translation inhibition, and others acted through transcript cleavage. Three *TaFes1s* (*TaFes1A-5A*, *TaFes1B-4A*, *TaFes1C-5D*) were targeted by one miRNA, three *TaFes1s* (*TaFes1A-5D*, *TaFes1C-5A*, *TaFes1C-5B*) were targeted by two miRNAs, and one *TaFes1* (*TaFes1A-5A*) was targeted by three miRNAs. None of the three *TaFes1s* homoeologues were targeted by the same miRNA, indicating that the regulation of *TaFes1* homoeologues by miRNAs would be different. This result provides useful information for further investigation of posttranscriptional regulation of *TaFes1s*.

### Expression profiles of *TaFes1s* under normal and abiotic stress condition

**Figure 5:**
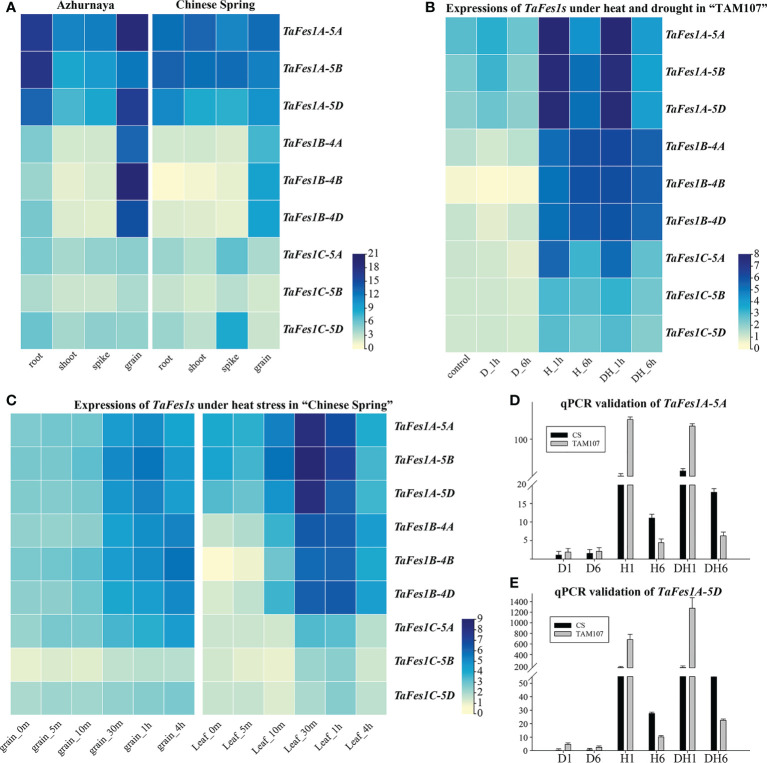
Expression profiles of *TaFes1s* in hexaploid wheat under normal and stress conditions. **(A)** Expression profiles of *TaFes1s* in wheat cultivar “Azhurnaya” and “Chinese Spring”, expression abundance is showed in tpm unit. **(B)** Expression profiles of *TaFes1s* in leaves of wheat cultivar “TAM107” at seedlings stage under heat and drought conditions. Expression abundance is shown as log_2_tpm. D_1h, drought stress for 1 hour, D_6h, drought stress for 6 hour, H_1h, heat stress for 1 hour, H_6h, heat stress for 6 hour, DH_1h, drought and heat stress for 1 hour, DH_6h, drought and heat stress for 6 hour. **(C)** Expression profiles of *TaFes1s* in wheat cultivar “Chinese Spring” at 15 days after anthesis under heat stress. Expression abundance is shown as log_2_tpm. The qPCR validation of *TaFes1A-5A* **(D)** and *TaFes1A-5D* **(E)** in wheat cultivar “Chinese Spring” and “TAM107” under heat and drought stress, where the expression level is check by three independent biological replicates and shown as 2^-ΔΔCt^. CS, Chinese Spring.

The transcription profiles of genes could provide important clues for illustrating the gene function, thus, the expression profiles of *TaFes1s* during wheat growth, development, heat stress, drought stress, and combined heat and drought stress were analyzed. Under normal condition, compared with *TaFes1Bs* and *TaFes1Cs*, all three *TaFes1As* were more highly and constitutively expressed across different tissues in both “Azhurnaya” and “Chinese Spring”, the three *TaFes1Bs* were likely preferred to be transcribed more in grain, and the three *TaFes1Cs* were constitutively but relatively lowly expressed (**Figure 5A**; **Supplementary Figure 2**). In wheat thermotolerant variety “TAM107” (**Figure 5B**), all *TaFes1s* were transcribed more in heat, and combined stress conditions, three *TaFes1As* were sharply responded to short time stress (H_1h and DH_1h), while three *TaFes1Bs* were stably highly expressed (H_1h, H_6h, DH_1h, and DH_1h). In wheat thermosensitive variety “Chinese Spring”, besides the fact that *TaFes1C-5B* were relatively lowly expressed, other *TaFes1s* showed similar expression patterns in grains (**Figure 5C**). In flag leaves, *TaFes1As* were transcribed most highly, and mainly under 30 minutes and 1 hour of heat stress. Finally, the expression patterns of *TaFes1A-5A* and *TaFes1A-5D* were checked by qPCR experiments and this confirmed that both genes mainly responded to short time stresses (**Figures 5D, E**).

### Roles of *TaFes1A-5A* and *TaFes1A-5D* in transgenic Arabidopsis

Based on the expression profiles of *TaFes1s* under normal and stress condition, we further selected *TaFes1As* for functional characterization. *TaFes1A-5A* and *TaFes1A-5D* were successfully cloned and transformed into an Arabidopsis thermosensitive *fes1a* mutant. For each gene, three independent T_3_ homozygous transgenic lines were finally obtained for further analysis.

**Figure 6:**
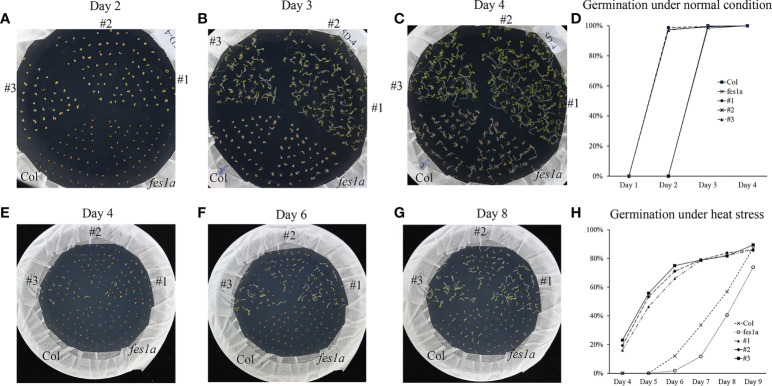
The seeds germination of *TaFes1A-5D* transgenic lines under normal and heat conditions. **(A–C)**. Dynamic germination of transgenic lines, wild type, and mutant seeds under normal condition (22/19°C, 16/8h). **(D)** Germination statistics calculated from **(A–C)** under normal condition. **(E–G)**. Dynamic germination of transgenic lines, wild type, and mutant seeds under heat stress condition (35/19°C, 16/8h). **(H)** Germination statistics calculated from **(E–G)** under normal condition.

It has been reported that the *fes1a* mutant was thermosensitive at seedling stage (Zhang et al., 2010), in this study, the mutant also showed defect in basal and acquired thermotolerance. However, overexpression of *TaFes1A-5A* and *TaFes1A-5D* could not complement thermal defect of *fes1a* mutants. Intriguingly, under normal condition, the seeds of transgenic lines germinated about 1 day earlier than wild type and *fes1a* mutant, and the wild type and *fes1a* mutant showed no difference (**Figures 6A–D**; **Supplementary Figure 3**). Under heat stress, the germination of transgenic seeds occurred significantly earlier than that of wild type and *fes1a* mutant. About 20% of transgenic seeds were germinated at day 4 of heat stress, and this ratio increased to 50% at day 5, while seeds of wild type and *fes1a* mutant started germination at this point (**Figure 6E–H**; **Supplementary Figure 3**). Thus, *TaFes1A-5A* and *TaFes1A-5D* could accelerate germination under both normal and heat stress conditions, but they could not rescue the thermotolerance defect of *fes1a* mutant at seedling stage.

## Discussion

The roles of Fes1s are largely known in humans and yeast, participating in protein quality control (Alberti et al., 2004; Gowda Naveen Kumar et al., 2013; Gowda et al., 2016; Rosenzweig et al., 2019). However, in plants, little is known about these genes. In Arabidopsis, three *Fes1s* were identified and attention has been paid to *AtFes1A*. Loss of *AtFes1A* resulted in thermotolerance defect, while the single knockout of a TFIIIB-related factor gene or a Bcl2-associated athanogene domain gene could suppress this defect (Zhang et al., 2010; Fu et al., 2015; Fu et al., 2019; Fu et al., 2020). The roles of *Fes1C* in ER and salt stress were also characterized in rice (Qian et al., 2021). While distribution, expression and roles of wheat *Fes1s* are still unknown.

In this study, we first characterized nine *Fes1s* in hexaploid wheat, and they are clearly classified into *Fes1A*, *Fes1B*, and *Fes1C* clades, presenting as triads. Further phylogenetic analysis found that except *Fes1s* in Arabidopsis, *Fes1As* and *Fes1Bs* were divergent in monocots but not in dicots, and *Fes1Cs* evolved into different subclades in monocots and dicots, indicating that *Fes1Cs* may have evolved later than *Fes1As* and *Fes1Bs*. Our results display the evolutionary relationship of *Fes1s* in monocots and dicots. However, the *Fes1s* in Arabidopsis always grouped together, and this is very interesting but based on our results we could not explain any further, additional functional analysis will be needed to determine whether their roles are conserved or not. In our functional characterization we found that two *TaFes1As* could not rescue the thermotolerance defect of Arabidopsis *fes1a* mutant at seedling stage, and this result may imply that the roles of *Fes1As* orthologs in these two species were different.

Next, we analyzed the sequence characteristics of *TaFes1s* and found that gene structures and protein motifs were highly conserved within each clade but distinct among clades. Similar degrees of sequence conservation were found in other chaperone gene families, such as wheat *HSP90s* (Wang et al., 2011; Lu et al., 2020), and *HSP101s* (Erdayani et al., 2020), and this conservation may indicate their similar biological roles. Consistently and finally, the functional characterization of *TaFes1A-5A* and *TaFes1A-5D* has revealed that both genes played similar roles in seed germination under normal and heat conditions in Arabidopsis.

The fates of homoeologous genes in polyploidy plants usually evolved into functional diversification, gene silencing, and the retention of original or similar functions. About 35.8% of the total genes in hexaploid wheat can be termed as triads (Appels et al., 2018). At transcriptional level about 30% of these triads showed biased expression patterns among each homoeologous gene under normal condition (Ramírez-González et al., 2018), and this ratio increased to about 64% under heat and drought conditions (Liu et al., 2015). The silencing of the homoeologues was usually found in different tissues and developmental stages (Bottley et al., 2006; Bottley and Koebner, 2008). The functional diversification of wheat homoeologous genes was observed in *wheat LEAFY HULL STERILE1* (Shitsukawa et al., 2007) and *Grain Weight 8* (Ma et al., 2019). The functional retention of wheat homoeologous genes was characterized in *TaEXPA1* (Hu et al., 2013b) and *SEPALLATA* (Shitsukawa et al., 2007). Sequence similarity and expression partitioning were important factors helping to deduce the functional conversation of homoeologous genes. Highly conserved sequences and expressions of wheat *SEPALLATA* led to the functional conservation of three homoeologs, and large differences in sequences and expressions of *wheat LEAFY HULL STERILE1* caused the three homoeologues to function differentially (Shitsukawa et al., 2007). However, using sequences and expressions for deduction maybe also easily misleading. The coding sequence of *TaEXPA1* homoeologues were high similar, and epigenetic modification led to expression divergence, but the three homoeologues displayed functional retention in Arabidopsis (Hu et al., 2013a; Hu et al., 2013b). Highly similar sequences and different expression patterns were also observed in wheat *Grain Weight 8* homoeologues, but these homoeologues were associated with different traits (Ma et al., 2019). Thus, understanding the functions of homoeologues requires extensive experiments.

## Data availability statement

The original contributions presented in the study are included in the article/**Supplementary Material**. Further inquiries can be directed to the corresponding author.

## Author contributions

YL conceived and designed the study. YL performed the analysis, drafted and revised the manuscript. MH and XL performed functional characterization. JW and RM contributed to the transgenic materials. AZ performed QPCR analysis. All authors contributed to the article and approved the submitted version.

## Funding

This study was mainly supported by the Natural Science Foundation of Hebei Province, China (C2020402026), and S&T Program of Hebei, China (22326303D).

## Acknowledgments

We are very grateful to Prof. Jian Liu of Shandong Normal University for kindly providing Arabidopsis *fes1a* mutant as a gift.

## Conflict of interest

The authors declare that the research was conducted in the absence of any commercial or financial relationships that could be construed as a potential conflict of interest.

## Publisher’s note

All claims expressed in this article are solely those of the authors and do not necessarily represent those of their affiliated organizations, or those of the publisher, the editors and the reviewers. Any product that may be evaluated in this article, or claim that may be made by its manufacturer, is not guaranteed or endorsed by the publisher.
