# Supplementary material for: Distribution, expression of hexaploid wheat Fes1s and functional characterization of two TaFes1As in Arabidopsis
Source: Front Plant Sci. 2022 Oct 17;13:1037989. doi: 10.3389/fpls.2022.1037989 (PMC9621618; doi:10.3389/fpls.2022.1037989)
Supplement: Supplementary file 1 [file DataSheet_1.pdf]

Distribution, expression of hexaploid wheat Fes1s and functional characterization of two TaFes1As in Arabidopsis

Yunze Lu[1]*, Mingran Ha[1], Xinming Li[1], Junzhe Wang[2], Ruirui Mo[2], Aihua Zhang[1]

1 Supplementary Figures and Tables

1.1 Supplementary Figures

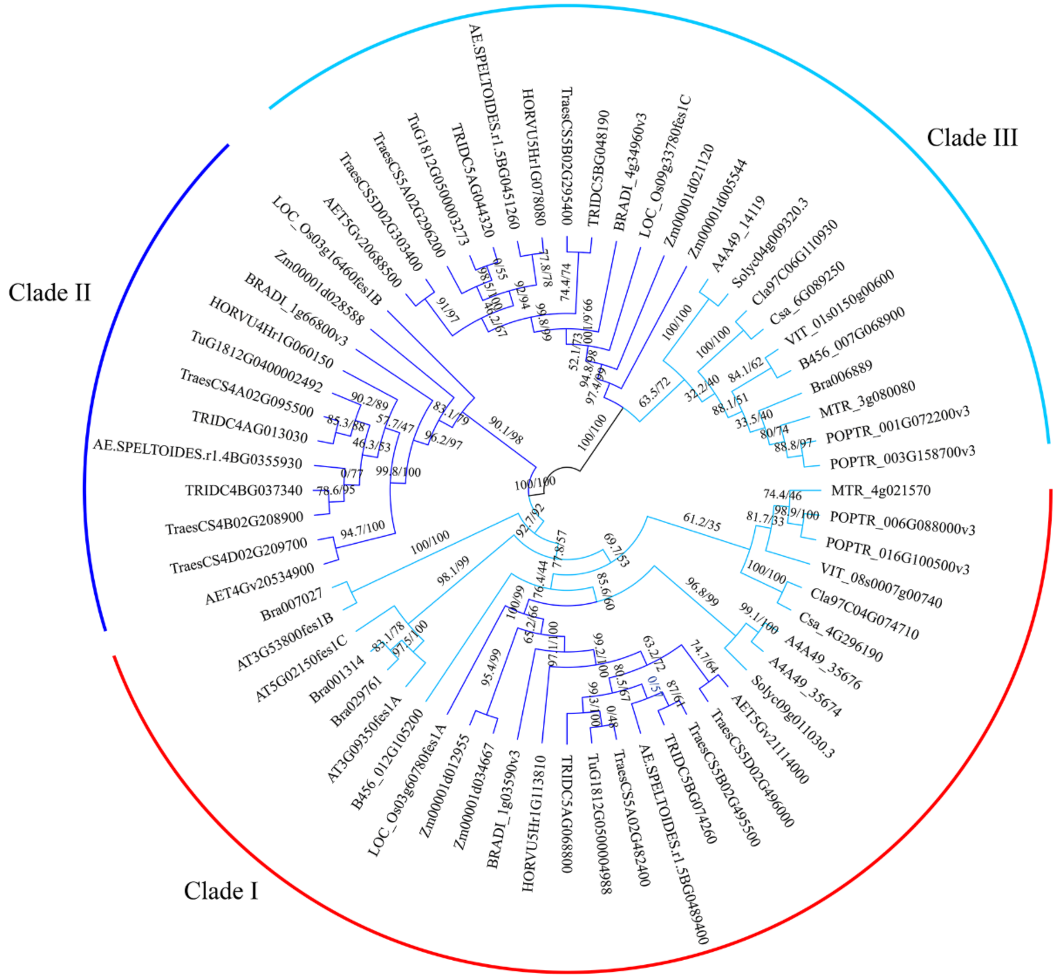

Supplementary Figure 1. Phylogenetic analysis of Fes1s in some dicot and monocot species. Protein sequences are aligned in MAFFT by “L-INS-i” algorithm. The phylogenetic maximum likelihood tree is built via IQ-TREE, the JTT+I+G4 substitution model is chose based on the Bayesian information criterion. Consistency of the phylogenetic estimate was evaluated with Ultrafast bootstraps as well as a Shimodaira-Hasegawa approximate likelihood ratio test (SH-aLRT) test (1000 replicates each). Species names are as following: monocots: AE.SPELTOIDES, *Ae.*

speltoides; AET, *Ae. tauschii*; BRADI, *Brachypodium distachyon*; HORVU, *Hordeum vulgare*; LOC, *Oryza sativa* ssp. *Japonica*; Ta, *T. aestivum*; TRIDC, *T. turgidum* ssp. *Dicoccoides*; Tu, *T. urartu*; Zm, *Zea mays*; Dicots: A4A49, *Nicotiana attenuata*; AT, *Arabidopsis thaliana*; B456, *Gossypium raimondii*; Bra, *Brassica rapa*; Cla, *Citrullus lanatus*; Csa, *Cucumis sativus*; MTR, *Medicago truncatula*; POPTR, *Populus trichocarpa*; Solyc, *Solanum lycopersicum*; VIT, *Vitis vinifera*.

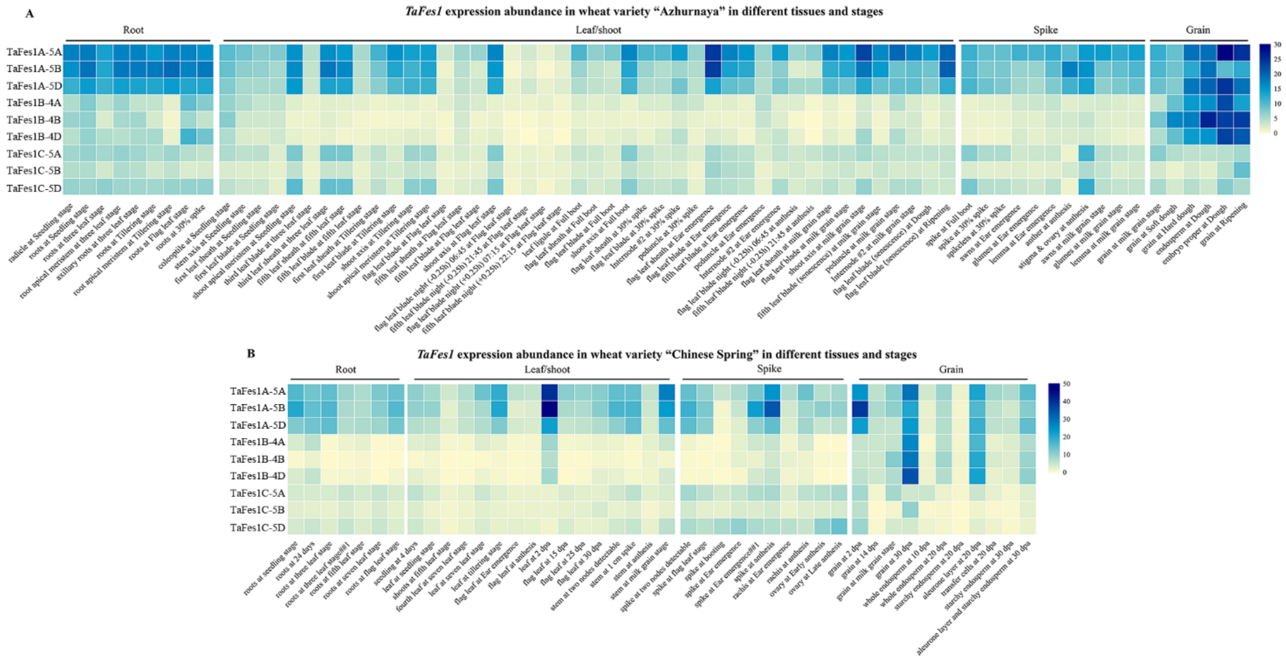

Supplementary Figure 2. Expression profiles of TaFes1s in wheat varieties "Azhurnaya" (A) and "Chinese Spring" (B). The expression data is obtained from Ramírez-González et al., 2018, Science. Expression value is shown in tpm unit.

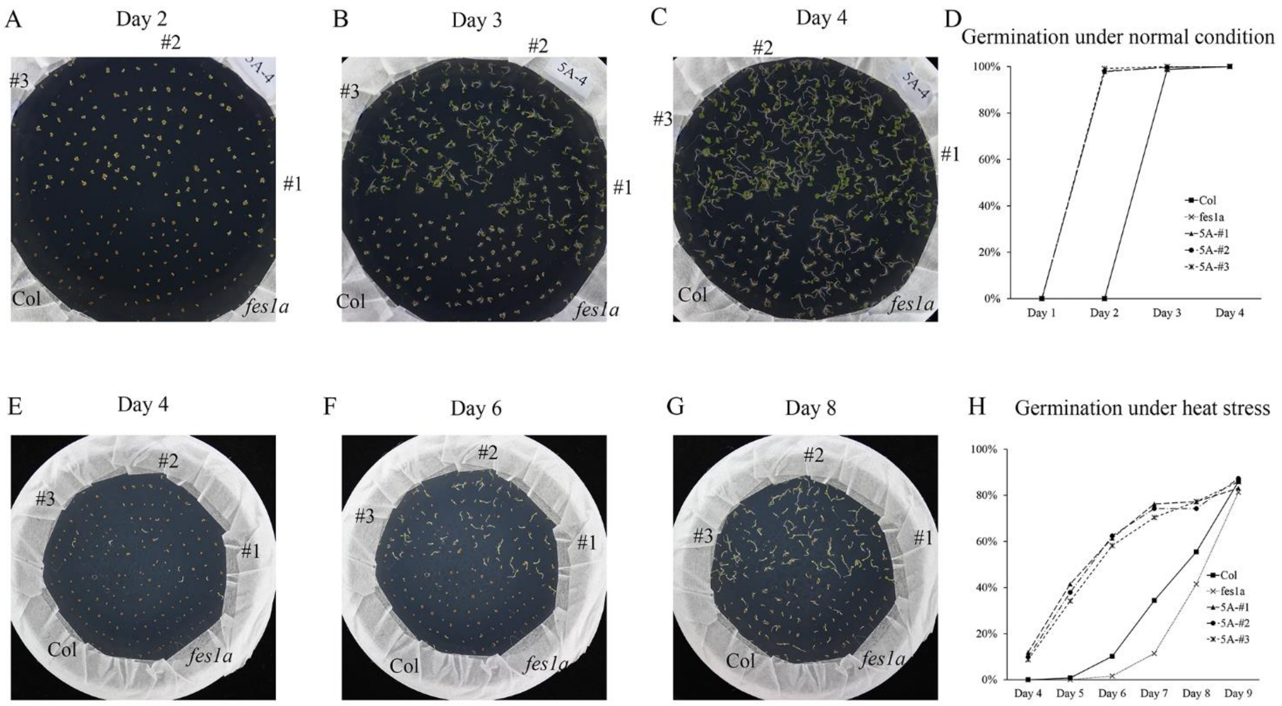

Supplementary Figure 3 The seeds germination of *TaFes1A-5A* transgenic lines under normal and heat conditions. (**A**)-(**C**). Dynamic germination of transgenic lines, wild type, and mutant seeds under normal condition (22/19℃, 16/8h). (**D**) Germination statistics calculated from (A) to (C) under normal condition. (**E**)-(**G**). Dynamic germination of transgenic lines, wild type, and mutant seeds under heat stress condition (35/19℃, 16/8h). (**H**) Germination statistics calculated from (E) to (G) under normal condition.

1.2 Supplementary Tables

Table S1. Sequences of primers used in this study.

Experiments	Primer name	Sequences (5'-3')
qPCR	Taactin-F	CTCCCTCACAACAACCGC
	Taactin-R	TACCAGGAACTTCCATACCAAC
	qTaFes1A-5A-F	GACCGTGACCGTCTTCCA
	qTaFes1A-5A-R	GCATCTGCCAAACTCAACC
	qTaFes1A-5D-F	CAAAGGAAAGTACACTCAAGTC
	qTaFes1A-5D-R	TCCAAACATCAGCTAACGA
gene cloning	TaFesA-5A-F	aaaaagcaggcttcATGGCCAAGGAAGGAGCAGG
	TaFesA-5A-R	agaaagctgggtcCGGTCCTAGCAGAAGGGGTG
	TaFesA-5D-F	aaaaagcaggcttcATGGCCAAGGATGGAGCAGG
	TaFesA-5D-R	agaaagctgggtcCGGCCCTAGCAGCAGGGGC
	attB1	GGGGACAAGTTTGTACAAAAAAGCAGGCT
	attB2	GGGGACCACTTTGTACAAGAAAGCTGGGT
Arabidopsis fes1a mutant identification	LB1	CAGAAATGGATAAATAGCCTTGCTTCC
	LP	CAAGTTCCTGACTTCTAGGATTGTTC
	RP	GTACGGACATTCATCTTGTAGTTGG

Table S2 Detail information of MEME motifs.

NO.	Width (a.a.)	NO. of sequences	LLR (Log Likelihood Ratio)	E-value	Consensus sequences
Motif-1	74	9	1525	1.80E-284	DIEDLLDELQVHVESIDIANDLHSIGGLVPVIRYLKNSNAEIRAKAAEV VSTVVQNNPKSQQLVMEANGLEPLV
Motif-2	116	6	1703	1.30E-269	ADRNVAAZJGLPRLLIHLVSSDDSGVREAALGGLLELAKDTTPGARNL LPDQDKLKDLLKGRIZGIRMMDPDDLDAAREERQLVDSLWKECYGEP SSLREEGLVVLPGEDAPZQPP
Motif-3	48	9	976	4.00E-171	NFTSDPSATARIKALGAISSLIRNNKPGLAAFRLENGHAALKDALGSE
Motif-4	159	3	1256	5.00E-108	MAKARPHSERSSHRRRNLLLAVALLSAALLLPAATASAAVAVAAEGD GENRSRGAATQWATGKDEGELAAEREAAGGGSVVEDDFAGGFGSLD SMLQWAIGNSDPGRLKEEAADVQKLSEDELLKRRHEIKDLMEKLKMP SDADLMKIAIADLNNASIS
Motif-5	113	3	925	4.80E-91	QKKAVFLITDLADFQLNSGSSGLPFLSERVFLKSVTDMLSKFDLDLQE KVLLAIRSLLKLPSTDATDLESCGLDSVLYRLGVQLEELPSEEQKEYAG EVDALRREVEMLFQQK
Motif-6	80	3	691	1.10E-80	KDGAGGGPDWNGLLKWSLAHGSDGTNPPRALSEEDRKWFMEAMQA NTVDVVGRMKEIAQVMKTPDDVLQSHGVTPENIEG
Motif-7	29	6	398	4.90E-43	GRFFPPLRRAPLPRPAPAPDEDSGPGKGT
Motif-8	47	3	389	1.70E-35	CSEEERRWLAEAVERHMAEDVVGRLREIALLMRTPLSVLEAQGITPE
Motif-9	15	6	237	5.40E-31	ARLQRKALHLTHYLL
Motif-10	36	2	211	2.10E-11	MAGDRMSWARLLKWSLSYMDGARPSRDIRWGEGKWR
Motif-11	15	3	124	1.80E-06	SAVFIWAAAEASVWC
Motif-12	15	3	117	2.00E-02	GGIMLLLGPAPGNSE
